# A Hint on Giving Iodide of Potassium

**Published:** 1874-04

**Authors:** 


### A HINT IN GIVING IODIDE OF POTASSIUM.

A useful hint is revived in the *British Medical Journal*, by Mr. Jos. P. McSweeny. He writes :

Sir James Paget was the first to call the attention of the medical profession to the following interesting fact, viz : that carbonate of ammonia greatly increases the therapeutic action of iodide of potassium. I have had extensive experience in the treatment of syphilis, and have tried it with the best results, and find that five grains of the iodide of potassium combined with three grains of carbonate of ammonia are equal to eight grains of the potassium salt administered in the ordinary way. The following case is a good example :

John ——, aged 50, consulted me about a sore situated on his left arm. There was a profuse discharge from it and the smell was intolerable. On asking him a few questions, I got the following history. He had been a married man, his wife having died a short time ago ; he had no children. Some years ago he contracted syphilis, and was treated by mercury, pushed to excessive salivation. The secondary symptoms had been well marked and the sore about which he consulted me was of eight months' standing. He consulted several surgeons and could get no relief. I ordered him five-grain doses of iodide of potassium combined with three grains of carbonate of ammonia. After taking a few tablespoonfuls of the bottle, the bad smell altogether disappeared, as a man told me who was sleeping in the same room ; at first he could not bear the smell, but after taking a few tablespoonfuls of the bottle he could detect no smell. The man remained under my care for about a month, and in that short time was perfectly cured and in very good health and spirits. I could publish five cases with almost similar results. I have also found it of the greatest service in the treatment of internal aneurism, by relieving the pain and helping to consolidate the tumor.

